# Social influences on the relationship between dissociation and psychotic-like experiences

**DOI:** 10.1017/S0033291724001405

**Published:** 2024-09

**Authors:** Charles Heriot-Maitland, Til Wykes, Emmanuelle Peters

**Affiliations:** 1 Department of Psychology, King's College London, Institute of Psychiatry, Psychology and Neuroscience, London, UK; 2 South London and Maudsley NHS Foundation Trust, London, UK

**Keywords:** dissociation, psychosis, psychotic like experiences, shame, social mechanisms

## Abstract

**Background:**

Shame is experienced as a threat to social self, and so activates threat-protective responses. There is evidence that shame has trauma-like characteristics, suggesting it can be understood within the same conceptual framework as trauma and dissociation. Evidence for causal links among trauma, dissociation, and psychosis thus warrant the investigation of how shame may influence causal mechanisms for psychosis symptoms.

**Methods:**

This study tested the interaction between dissociation and shame, specifically external shame (feeling shamed by others), in predicting psychotic-like experiences (PLEs) six months later in a general population sample (*N* = 314). It also tested if social safeness moderates these effects. A longitudinal, online questionnaire design tested a moderation model (dissociation-shame) and a moderated moderation model (adding social safeness), using multiple regressions with bootstrap procedures.

**Results:**

Although there was no direct effect of dissociation on PLEs six months later, there was a significant interaction effect with shame, controlling for PLEs at baseline. There were complex patterns in the directions of effects: For high-shame-scorers, higher dissociation predicted higher PLE scores, but for low-shame-scorers, higher dissociation predicted lower PLE scores. Social safeness was found to significantly moderate these interaction effects, which were unexpectedly more pronounced in the context of higher social safeness.

**Conclusions:**

The results demonstrate evidence for an interaction between dissociation and shame on its impact on PLEs, which manifests particularly for those experiencing higher social safeness. This suggests a potential role of social mechanisms in both the etiology and treatment of psychosis, which warrants further testing in clinical populations.

Evidence for dissociative and social pathways in psychosis (Heriot-Maitland, Wykes, & Peters, 2021) and for the traumatic/dissociative properties of shame (Dorahy et al., 2017; Kouri, D'Andrea, Brown, & Siegle, 2023; Matos & Pinto-Gouveia, 2010) supports a theoretical framework in which shame, a ‘threat to social self’, could influence dissociative processes as a potential causal mechanism for psychosis symptoms. In line with social rank theory (Gilbert, 1992), shame is considered to be a negative social ranking experience that threatens human survival needs for acceptance and connection. Therefore, it may be that the presence of shame, particularly external shame (feeling shamed by others), can activate threat-responses, drive dissociative processes, and maintain vulnerability to psychotic-like experiences (PLEs). Previously, shame has typically been researched as a consequence of psychosis (Watson, Corrigan, Larson, & Sells, 2007) or as related to anxiety and depression in the context of psychosis (Keen, George, Scragg, & Peters, 2017; Michail & Birchwood, 2013; Upthegrove, Ross, Brunet, McCollum, & Jones, 2014; Wood & Irons, 2016). However, bar a few notable exceptions (McCarthy-Jones, 2017), the role of shame as a causal mechanism in psychosis has received little attention, perhaps because such a major focus of the last decade has been on the role of childhood adversity and trauma in psychosis, via dissociation mechanisms (Varese et al., 2012). This may have overshadowed the idea that shame itself may be experienced as traumatic, with dissociative properties of its own.

Like trauma memories, shame experiences and memories may be highly threatening to an individual, particularly in relation to their social position (rank) and inclusion, triggering dissociative processes to avoid or regulate these overwhelming feelings. In an experimental study, Kouri et al. (2023) found that shame-related memories generated dissociative state responses, and that the degree of people's dissociation was moderated by their level of experiential avoidance (conscious attempts to avoid the feeling of shame). This supports the idea that dissociative states may function as a strategy to protect against feelings of shame. This builds on earlier experimental work by Dorahy et al. (2017) that also demonstrated a causal relationship between shame and dissociative states. Such experimental studies have not yet been conducted in the psychosis literature; however, because of the relevance to psychosis of both variables – shame and dissociation – this is an area of clear potential and research interest. In the case of voice-hearing, specifically, McCarthy-Jones (2017) has proposed a model of the ‘hallucinogenic’ properties of shame, and presents testable hypotheses around the relationship between shame and voices. However, the influence of shame may extend to processes that drive other positive symptoms, such as delusions and paranoia. Indeed, a recent systemic review of shame and psychosis (Carden, Saini, Seddon, Watkins, & Taylor, 2020) identified more studies that found a positive association between shame and paranoia than between shame and voices. One study provided evidence for the role on shame in amplifying the relationship between stressful life events and paranoia (Johnson et al., 2014). Hence, it is possible that shame may contribute to processes that are driving a range of experiences on the psychosis continuum.

The current study investigates how the interplay of these key processes – dissociation and shame – impact upon psychotic-like experiences (PLEs) more broadly, using a unidimensional scale that assesses PLEs in the general population, the *Transpersonal Experiences Questionnaire* (TEQ) (Heriot-Maitland et al., 2023). Specifically, this study tests a dissociation–shame interaction model, whereby dissociative traits are hypothesized to become magnified or maintained through their interaction with external shame. In this model, there is a conceptual distinction between dissociative traits, which may be pre-existing / historical, and the acute dissociative (state) reactions that were found, in the earlier experimental research, to be elicited from (current) shaming experiences. One of the limitations of previous studies on dissociation and psychosis is that many have used cross-sectional designs, which cannot determine direction of causality (Carden et al., 2020). This study aimed to address this gap by using a longitudinal design. The study recruited from the general population, which is a commonly used recruitment strategy for studying mechanisms in psychosis, due to the evidence for a continuum of psychosis throughout the population (Peters et al., 2016; van Os, Linscott, Myin-Germeys, Delespaul, & Krabbendam, 2009).

Social safeness (pro-social, affiliative experiences) may also play a protective role in buffering or regulating these interacting processes. Social safeness is the term used by Gilbert (2009) to refer to the soothing qualities of caring and attachment experiences, which link to feelings of wellbeing, safeness, and social connectedness. Social support is found to have an important role in protecting against stress generally (Hostinar & Gunnar, 2015) and against psychosis symptoms more specifically (Norman et al., 2005). According to Gilbert's model of social mentalities (Gilbert, 2009, 2014) and neuroscientific models of emotion systems (Depue & Morrone-Strupinsky, 2005), social safeness and threat are linked to different systems, with distinct functions and physiologies (in the same way that para-sympathetic and sympathetic nervous systems are functionally and physiologically distinct). Hence, in this study, these two social mechanisms – external shame (a threat to social self) and social safeness – are explored as distinct, each with potentially different influences on dissociative and psychotic processes. As distinct mechanisms, it is plausible that both social threat and safeness systems could be activated in parallel; and hence, why a three-way interaction (dissociation × shame × social safeness) is tested in a moderated moderation model.

Specifically, the following hypotheses were tested:

1. Dissociative traits will predict PLEs six months later (Fig. 1a).
2. External shame will moderate the longitudinal relationship between dissociative traits and PLEs (Fig. 1b).
3. Social safeness will moderate the magnitude of the moderating effect of shame on the longitudinal relationship between dissociative traits and PLEs (Fig. 1c).

**Figure 1.**
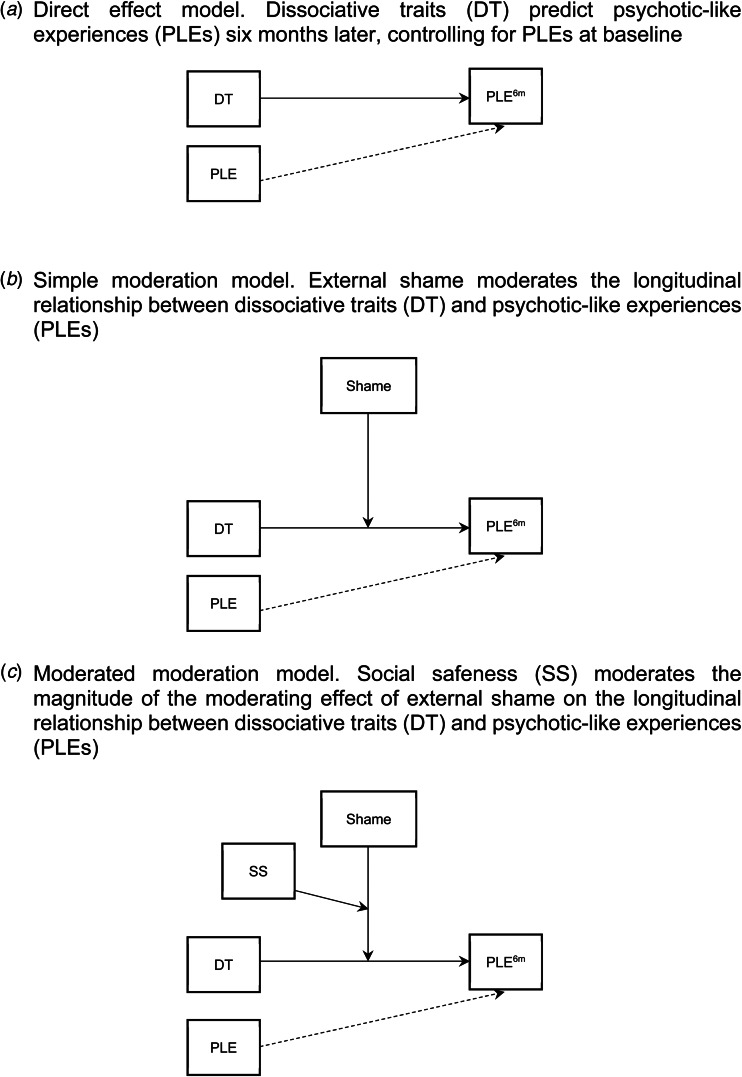
Models tested.

## Methods

### Design and participants

This study used a longitudinal, online questionnaire design with two time points (six months apart) to test three models (Fig. 1). The sample was recruited from the general population, with the only exclusion criterion being that participants should not be under 18 years old. This web-based design has been employed previously in the general population to research PLEs and predictive factors (Freeman et al., 2005; Oliver, McLachlan, Jose, & Peters, 2012).

### Measures

#### Demographics

Age, gender, ethnicity, education level, first language, and contact with mental health services.

#### Psychotic-like experiences (PLEs)

The *Transpersonal Experiences Questionnaire* (*TEQ*) (Heriot-Maitland et al., 2023) is a 19-item self-rated scale measuring psychotic-like, anomalous, experiences. A broad range of experiences are included, across ideational and perceptual domains, from ‘seeing/hearing/smelling things’, through ‘messages and hints’ and ‘feeling monitored or watched’, to ‘thought interference’ and ‘time distortion’, forming a single unidimensional scale. The items are de-coupled from pathology, intended for research in non-clinical populations. The full TEQ can be accessed directly (open access) here: https://doi.org/10.1111/papt.12445. Items are rated Yes/No according to whether an individual has had the experience ‘in the past 7 days’. Total scores range from 0 to 19. The TEQ has been validated for use in the general population and has good internal consistency (Cronbach's *α* 0.85).

#### Dissociative traits (DT)

The *Revised Dissociative Experiences Scale* (*DES-II*) (Carlson & Putnam, 1993) is a 28-item self-report scale measuring trait dissociation. Items are rated as percentages (in 10% increments from 0% = never to 100% = always) according to how often people have the experience (‘generally in life’). DES-II captures feelings of depersonalization, derealization, absorption, and amnesia. Total scores range 0–100. It is the most widely used measure of dissociation and has high internal consistency (Cronbach's *α* 0.90).

#### Shame

The *Other as Shamer Scale* (*OAS*) (Goss, Gilbert, & Allan, 1994) is an 18-item self-report measure of external shame. Items are rated on a five-point scale according to the frequency of evaluations about how others judge the self (0 = never to 4 = almost always). Items tap socially based concerns, such as ‘I feel other people look down on me’ and ‘other people see me as somehow defective as a person’, making it a suitable measure of threat to social self in this study. Total scores range 0–72, and it has high internal consistency (Cronbach's *α* 0.92).

#### Social safeness (SS)

The *Social Safeness and Pleasure Scale* (*SSPS*) (Gilbert et al., 2009) is an 11-item self-report scale measuring the extent to which people experience their social worlds as safe, warm, and soothing. Items are rated on a five-point scale (1 = almost never to 5 = almost all the time). Items tap feelings of belonging, acceptance, such as ‘I feel connected to others’ and ‘I feel a sense of warmth in my relationships with people’. Total scores range 11–55, and it has high internal consistency (Cronbach's *α* 0.92).

### Procedure

The study received ethical approval by the King's College London PNM Research Ethics Subcommittee (ref: PNM/14/15-26). Participants were recruited in 2015–2016 via adverts on websites and email lists, including King's College London (www.kcl.ac.uk) and www.experimatch.com. Upon responding and providing informed consent, participants accessed the questionnaires, hosted on Bristol Online Surveys (www.onlinesurveys.ac.uk).

Participation was remunerated through a prize draw (1^st^ £100, 2^nd^ £50, & 3^rd^ £25 prizes). Invitation for follow-up retest was offered to all participants, with an additional prize draw for those who completed this. Those opting-in were asked to leave their email address, so that a link to the retest questionnaire could be sent 6 months later. Participants completed all questionnaires on both occasions. The two sets of responses (baseline and follow-up) were matched by participant identification codes.

### Data analysis

In testing distribution normality, histogram and Q–Q plots of dependent variable (TEQ^6m^) demonstrated positive skewness, and a Shapiro–Wilk's test confirmed non-normality (*W* = 0.719, *p* < 0.001). Therefore, non-parametric tests were used for the correlation analyses (Spearman's rho). For the regression analyses, in accordance with Russell and Dean (2016), the data were not log-transformed, but instead analyzed using the recommended method of bootstrapping with the original (positively-skewed) TEQ^6m^ scores. Correlation analyses were used to check the stability of DES scores over time, as required for its use as a ‘trait’ measure, and secondly, to check whether DES and TEQ were empirically distinct, as required for the testing of moderation models. A series of multiple linear regressions were then used to test the models. All analyses were conducted in SPSS 24 (IBM, 2016). The moderation models (B and C) were run in the PROCESS v3 macro (Hayes, 2017) for SPSS, using 5000 bootstrap samples. To test moderation, PROCESS uses a simultaneous entry method, as opposed to a hierarchical (i.e. entry in steps) method, with each of the models A, B, C being tested independently as a ‘stand-alone’ analysis. Despite using simultaneous entry, PROCESS is programmed to yield an *R^2^* change value within each moderation model, where *R^2^* change is mathematically identical to the squared semi-partial correlation for the interaction term (Hayes, 2017); hence why it is not necessary to calculate an *R^2^* change by comparing incremental improvements in fit between hierarchically different models (Hayes (2017), p. 290). The directions of effects were then interpreted visually using interaction plot graphs.

## Results

### Longitudinal sample selection

A sample of *n* = 314 provided responses at both baseline and 6-month follow-up. The number entering at baseline was 544, meaning that 58% comprised the final longitudinal sample. To examine selection bias in the sample, comparisons between completers (*n* = 314) and non-completers (*n* = 230) were tested using chi square tests (demographic data) and Mann–Whitney tests (questionnaire data; data were non-normally distributed). See comparison table in Appendix 1 (supplementary material). There were no significant differences found between completers and non-completers on gender, language, mental health service use, PLEs, external shame, or social safeness. However, completers were significantly older (35% over 30 years, compared to 24.8%), more highly educated (72.6% degree or higher, compared to 64.3%), more likely to be of a white ethnic background (80.6%, compared to 69.1%), and had lower dissociation scores (mean 13.78, compared to 16.74) than non-completers.

### Demographics

In the final sample, most participants were women (80%), in the 18–29 age range (65%), white (white British 52%, white other 29%), and highly educated (with 73% to degree level), and the main first language was English (79%). 57% of participants had never been in contact with services regarding mental health, and of those who did report contact, 34% had received a diagnosis (26% mood disorder, 1% psychotic disorder, 7% other) and 9% had not.

### Correlations

The directions of all correlations were as expected (Table 1), with positive correlations between the measures of PLEs (TEQ), dissociative traits (DES) and external shame (OAS), and negative correlations between each of these and the measure of social safeness (SSPS). Due to multiple correlations, *p* values for detecting significance were adjusted to *p* < 0.01. All correlations were statistically significant at *p* < 0.01, with one exception, SSPS and TEQ^6m^, which had a non-significant negative correlation. The dissociative traits measure (DES) was re-administered at 6 months to check stability of scores over time. A strong positive correlation between DES scores at both timepoints (*r* = 0.78, *p* < 0.01) confirmed stability. The correlations between DES and TEQ at both time-points were only moderate (*r* = 0.57 (baseline) and *r* = 0.55 (6 m follow-up), *p* < 0.01), meaning that the two variables of dissociative traits and PLEs could be regarded as empirically distinct, as is required for the testing of moderation models.

**Table 1.** Means, standard deviations, medians, interquartile ranges, and Spearman's inter-correlations of variables at baseline and 6 months (*n* = 314)

	*Mean*	*SD*	*Med*	*IQR*	TEQ	DES	OAS	SSPS	TEQ^6m^
TEQ	2.45	2.99	1.00	4.00					
DES	13.18	10.57	10.00	11.88	0.57*				
OAS	22.72	12.61	21.50	16.00	0.29*	0.36*			
SSPS	37.75	8.78	38.00	12.00	−0.15*	−0.22*	−0.52*		
TEQ^6m^	2.11	3.02	1.00	3.00	0.70*	0.47*	0.27*	−0.10	
DES^6m^	12.08	10.33	9.29	11.52	0.52*	0.78*	0.36*	−0.22*	0.55*

TEQ, Transpersonal Experience Scale; DES, Revised Dissociative Experiences Scale; OAS, Other as Shamer Scale; SSPS, Social Safeness and Pleasure Scale.

*p < 0.01.

### Model A: direct effects

The results in Table 2A show that DES did not significantly predict TEQ^6m^ scores at 6 months, controlling for TEQ scores at baseline (*b* = 0.021, *t*(311) = 1.636, *p* = 0.103), meaning that the first hypothesis (1) was not supported. However, the absence of a direct effect does not prevent subsequent testing of moderation effects (Hayes, 2017), and therefore testing models B and C for moderation effects could proceed.

**Table 2.** Regression analysis results (*n* = 314): testing models A, B & C, with TEQ^6m^ as dependent variable

	Coeff. (*b*)	*SE*	*t*	*p*
Model A: direct effects				
Constant	0.129	0.188	0.686	0.493
DES	0.021	0.013	1.636	0.103
TEQ	0.692	0.046	15.082	**<0.001
	*R^2^* = 0.531, *MSE* = 2.072, *F*(2311) = 176.081, *p* < 0.001			
Model B: simple moderation				
Constant	0.506	0.387	1.308	0.192
DES	−0.038	0.026	−1.458	0.146
OAS	−0.009	0.016	−0.586	0.558
DES × OAS	0.002	0.001	2.313	*.021
TEQ	0.690	0.046	15.049	**<0.001
	*R^2^* = 0.544, *MSE* = 4.199, *F*(4309) = 92.273, *p* < 0.001			
Model C: moderated moderation				
Constant	0.623	1.783	0.349	0.727
DES	0.003	0.111	0.026	0.980
OAS	0.026	0.057	0.447	0.655
DES × OAS	−0.004	0.003	−1.269	0.205
SSPS	−0.003	0.041	−0.073	0.942
DES × SSPS	−0.001	0.003	−0.509	0.611
OAS × SSPS	−0.001	0.001	−0.669	0.504
DES × OAS × SSPS	0.0002	0.0001	2.393	*0.017
TEQ	0.710	0.045	15.928	**<0.001
	*R^2^* = 0.584, *MSE* = 3.887, *F*(8305) = 53.441, *p* < 0.001			

*p < 0.05; **p < 0.001.

### Model B: simple moderation

**Figure 2.**
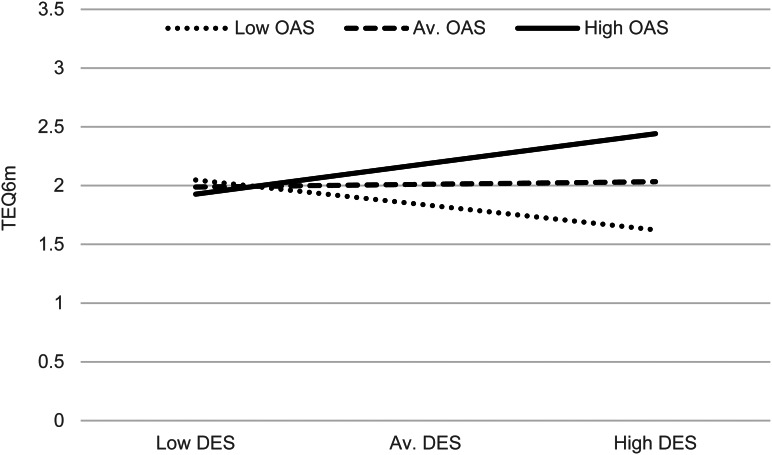
Plots of the interaction effects (DES × OAS) on TEQ^6m^.

Table 2B shows that OAS significantly moderated the relationship between DES and TEQ^6m^, controlling for TEQ at baseline (*b* = 0.002, *t*(309) = 2.313, *p* = 0.021), with the interaction term (DES × OAS) explaining a small but significant amount of variance in TEQ^6m^ (*R^2^* change = 0.008, *F*(1309) = 5.350, *p* = 0.021). The direction of these effects is illustrated by the plots in Fig. 2, which show the interaction slopes at different levels of OAS. For people with high OAS scores (the top slope), the higher the DES score, the higher the TEQ^6m^ score. This is the expected direction (in hypothesis 2). However, the other plots show that for people with average OAS scores, there is only a marginal positive relationship between the two, and for low OAS scorers, there is a negative relationship (i.e. the higher the DES score, the lower the TEQ^6m^ score). Although there is an overall significant interaction effect, the interaction plots reveal that the direction/s of this effect are more complex than first anticipated.

### Model C: moderated moderation

**Figure 3.**
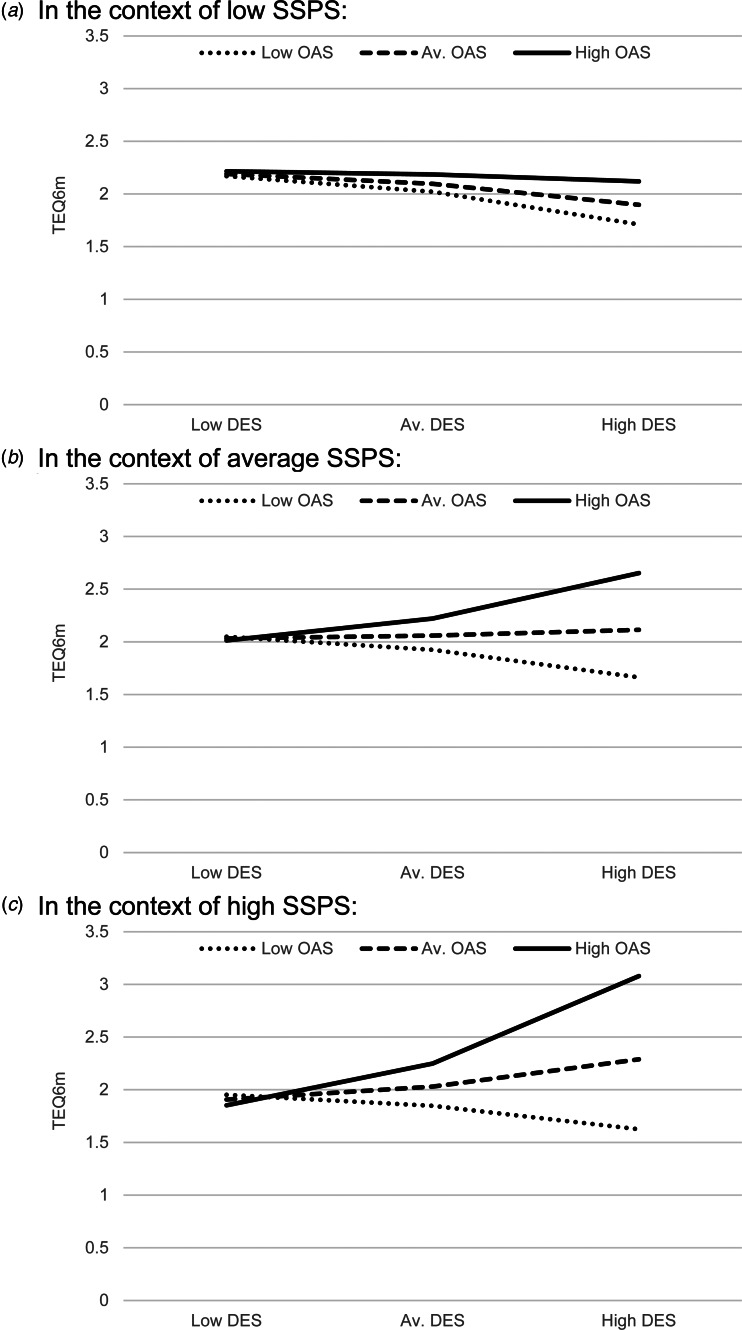
Plots of the interaction effects in the context of different levels of SSPS.

Table 2C shows that SSPS significantly moderated the magnitude of the moderating effect of shame on the relationship between DES and TEQ^6m^ (*b* = 0.0002, *t*(305) = 2.393, *p* = 0.017). The three-way interaction term (DES × OAS × SSPS) again explained a small but significant amount of variance in TEQ^6m^ scores (*R^2^* change = 0.008, *F*(1305) = 5.276, *p* = 0.017). Figure 3 displays the three-way interaction plots, showing how the above DES × OAS interaction operates at different levels of SSPS. The plots reveal that the interaction only seems to be occurring in the context of average and high SSPS scores. In the context of low SSPS scores (top graph), the different levels of OAS do not impact on how DES scores are influencing TEQ^6m^ scores. So, again, although the three-way interaction effect is statistically significant, the plots reveal more complexity in the directions of effects, and actually showed SSPS to have a moderating effect in a direction opposite to what was expected (hypothesis 3), in that higher SSPS scorers had more pronounced DES × OAS interactions.

## Discussion

The findings show evidence of an interaction effect between dissociation and external shame, a social rank threat, in predicting PLEs six months later in a general population sample. This supported the moderation hypothesis (2). This interaction effect was significant despite the absence of a direct effect (i.e. dissociation predicting PLEs at six months, which was not supported [hypothesis 1]). The interaction plots showed interesting differences in the predictive effects of dissociation on PLEs at different levels of shame: At high levels of shame, higher dissociation predicted more PLEs; at average levels, there was no predictive effect; and at low levels of shame, higher dissociation predicted fewer PLEs. Social safeness, which was negatively correlated with all other variables, had a significant moderating influence on the dissociation-shame interaction. While a significant effect was hypothesized (hypothesis 3), the directions were different from those predicted: At high levels of social safeness, the dissociation-shame interaction was *more* pronounced, and in the context of low social safeness, there was no dissociation-shame interaction in predicting PLEs.

### No direct effect of dissociation on PLEs

The finding that dissociation did not predict PLEs directly at six months was at odds with some of the existing literature. For example, in a meta-analysis of 19 (mostly cross-sectional) studies, Pilton, Varese, Berry, and Bucci (2015) reported a significant positive relationship between dissociative experiences and voice-hearing. A potential reason for this unexpected finding is that some with higher levels of dissociation did not provide a 6-month follow-up. Discrepancy in findings might also be due to a number of differences in design; the current study tested a greater range of PLEs than just voice-hearing (only one of 19 TEQ items relates to voices), and also used a longitudinal design, controlling for PLEs at baseline, whereas the majority of studies reviewed by Pilton et al. (2015) were cross-sectional. Looking only at this study's cross-sectional data, there were significant correlations of moderate strength between dissociation and PLEs, so cross-sectionally, relationships were consistent with Pilton et al. (2015). This highlights the importance of longitudinal designs in differentiating relationship effects from directional effects.

### The interaction effect of dissociation-shame on PLEs

The second finding, that dissociation predicts PLEs at follow-up when in interaction with shame, suggests that even if dissociation may itself not predict PLEs, that further factors may increase its predictive strength. At high levels of external shame, high levels of dissociation led to higher levels of PLEs. This supports the dissociation-shame interaction model. The more complex finding to interpret is why, at low levels of shame, high levels of dissociation would lead to lower levels of PLEs. To interpret this, it may be helpful to distinguish between reasons for why dissociation might occur: in some situations, dissociation may occur in response to shame and ‘threats to social self’ (to avoid/regulate shame emotions, as described in the introduction), whereas in others, it may occur in response to physical threats (to avoid/regulate other emotions such as fear, anger, etc.). Hence, it might be helpful to hold in mind a distinction between ‘shame-driven-dissociation’ and ‘non-shame-dissociation’. One possibility is that only those with shame-related dissociation had dissociative processes that led to PLEs, and those with other drivers of dissociation did not. The suggestion here would be that there is something specific about the *social* nature of shame-based threats that is relevant for understanding PLEs. And when dissociation occurs for other (non-social) reasons, this may actually lower the likelihood of PLEs, perhaps through lowering emotional arousal more generally.

Another possibility is that PLEs could be the result of a double impact of different threat-sources on dissociative processes – the presence of (pre-existing) dissociative traits, combined with current shame – which could overload the dissociation, leading to increased mental divisions, sensory intrusions, and PLEs. In this case, the suggestion would be that it is the intensity of the *combined* threat sources (historical and current), rather than the *social* nature of threat *per se*, that leads to PLEs. Either way, there is still something about the absence of shame that seems to lower the likelihood of PLEs, and it may be that with no shame, dissociation may actually be more successful at doing its job of reducing arousal.

### Social safeness influences on the interaction effect of dissociation-shame on PLEs

The third finding was that social safeness moderates the dissociation-shame interaction effect on PLEs, but not in the direction predicted. Social safeness was expected to have a down-regulating effect on the interaction, but the plots showed that those who felt more socially connected were *more* influenced by differing shame levels, in terms of the predictive effects of dissociation on PLEs. Interpretation can, again, start by considering the possible distinction between shame-driven-dissociation and non-shame-dissociation. For non-shame-dissociators (i.e. the bottom ‘low shame’ lines of Fig. 3), there is no change in the slopes across all three graphs. So social safeness does not affect the job that dissociation is doing for them – perhaps because their dissociation is not linked to socially-based threats. For shame-dissociators, however, the social safeness level has a considerable impact – perhaps because their dissociation is serving a specifically social function, i.e. to regulate shame feelings. It may be that when people are less connected, the experience of external shame does not have such threatening (trauma-like/dissociative) properties. These people may be more isolated, so what they believe other people think of them is not such a concern or threat to their ‘social self’, and less likely to activate threat-protective responses. So shame may be present, but not in such a socially threatening way. In the context of high social safeness, however, where people are more socially connected, engaged and integrated, feeling shamed by others is potentially more threatening; and hence more likely to have traumatic properties. Another interpretation, bringing a perspective from attachment theory, is that the observed pattern of combined high social safeness and social threat (together) may be akin to ‘disorganized attachment’, which is a specific pattern of social disorientation and conflict that is often implicated in pathways to psychosis (Barker, Gumley, Schwannauer, & Lawrie, 2015).

Other influences on the directions of results may come from the measurement tools themselves, particularly if there is overlap between items. For example, one of the SSPS items (4: ‘I feel part of something greater than myself’) may tap connectedness in more of a spiritual sense, which might overlap with PLEs (e.g. TEQ 14: ‘have you had an experience of a loss of your individual identity and a sense of being part of some greater whole that extends far beyond you?’). However, as SSPS and TEQ were negatively correlated (SSPS-TEQ significantly, and SSPS-TEQ^6m^ non-significantly; Table 1), this is not likely to have confounded the results.

One final consideration in interpreting the three-way interaction is to think about the effects that social safeness might have on how a person relates to their PLEs. Feeling socially safe could itself represent a ‘secure base’ (in attachment theory language) from which one is more able to safely access and open their mind to diverse experiences, as opposed to suppressing and avoiding them, which is what could happen when feeling less safe. Hence these results could reflect a greater awareness, articulation, or reporting of PLEs among people scoring high on dissociation who feel socially safe, compared to those who do not. Feeling socially unsafe might lead to more attempts to shut down PLEs, which does not mean they are not present – just actively avoided. The measurement of PLEs was unrelated to emotional tone (positive, negative, distressing, etc.), so it could be that socially safe participants had more openness and readiness for PLEs, which may indeed be welcomed.

### Research and clinical implications

This study has implications for fine-tuning models of mechanisms in pathways from social-rank threat to psychosis (via dissociation). It suggests that future studies could be designed to test a modified, updated model on the role of dissociation in psychosis, with and without external shame. The possibility that dissociation, on its own, may not lead to PLEs (and may even reduce them via lowered threat arousal), but may lead to PLEs when combined with, or activated by, shame, is important for focusing attention toward social influences on these mechanisms. More generally, understanding shame within a similar conceptual framework as traumatic and dissociative processes, could prove a fruitful direction for future psychosis research. This is consistent with studies by Matos and Pinto-Gouveia (2010) and Dorahy et al. (2017), who have investigated the ‘trauma-like’ qualities of shame, and also aligns with Gilbert's evolutionary analysis, which links social rank experiences to evolved threat-protection mechanisms (Gilbert, 2000, 2014). Testing whether internal shame shows similar relationships to external shame would be interesting for further fine-tuning these models.

These processes also require testing within clinical populations with psychosis to see whether these patterns are consistent among those with a psychosis-related diagnosis, who are likely in many cases to be experiencing higher levels of dissociation (Carlson & Putnam, 1993) and shame (Wood & Irons, 2016) than the current sample. While further research is needed in these populations, the already considerable evidence for a psychosis continuum throughout the population (Peters et al., 2016; van Os et al., 2009) strengthens the applicability of these results in understanding psychosis, whether clinically diagnosed or not.

These findings also have implications for the treatment of psychosis, for example, in focusing attention on social factors, not only as consequences of psychosis but also as causal mechanisms. Interventions that specifically target shame, especially among high dissociators, could potentially have an impact on PLEs. Also, as the three-way interactions showed, the impact of targeting shame-reduction could be even more pronounced within the context of social safeness. If these findings were replicated in psychosis samples, there may be implications for creating therapeutic experiences of social safeness first, before targeting shame. These implications go beyond the therapy room, for instance, in supporting peer initiatives, like Hearing Voices groups and other opportunities to foster social connection.

### Strengths, limitations, and future research

There are several strengths and limitations of this study. A strength is the longitudinal design which is better able to test the direction and causal relationships between variables. Online designs can help with achieving better-powered longitudinal studies; however, they are not without limitations. For example, there was a lack of quality control over the participant responses (e.g. effort, concentration, time taken). This study attempted some control by giving participants the expected completion time; however, it was not possible to check the actual time taken. Another limitation was the sample, which was not representative of the general population; namely, with an overrepresentation of students (highly educated, aged 18–29) and women. There was also evidence of some selection bias, in that those completing follow-ups were older, more educated, more likely to be of a white ethnic background, and, importantly, had lower dissociation scores. Therefore, as mentioned above, a potential reason there was no direct effect of dissociation on PLEs, as had been predicted, is that those with higher levels of dissociation did not complete the follow-up, also possibly limiting the robustness of our other findings. There were other limitations regarding the measures used. For example, there may have been some overlap in items of feeling connected on SSPS (socially) and TEQ (spiritually), as well as between DES and TEQ, which both contained an item on hearing voices. There was also the limitation of using a general population sample, rather than a clinical sample, meaning that the clinical implications discussed are speculative until further research is carried out.

Future research should seek to recruit more representative longitudinal samples from the general population, with an emphasis on limiting attrition at the follow-up stage, and adapt these hypotheses for testing within clinical populations with psychosis. Further clinical research is also indicated to test these findings within the context of interventions that aim to reduce external shame in contexts of social safeness. This might include structured therapy interventions but also extends to community-based social interventions offered by peer supporters and organizations such as the Hearing Voices Network and Spiritual Crisis Network. Evaluating these socially-based approaches, with both qualitative and quantitative methods, could help to enhance our understanding of the role of social factors in both driving and helping psychotic phenomena. Another suggestion for future research would be to look specifically at emotional dimensions of PLEs; for example, the impact of these social mechanisms on PLE-related distress, as opposed to just the occurrence and variety of PLEs. Finally, future studies could include additional variables of high relevance; e.g., past or current traumas, adversities, threats, and other causes of dissociative processes.

## Conclusions

This study suggests that social factors, such as feeling shamed by others and feeling safe/connected with others, can contribute to the development of psychosis symptoms. This has implications for understanding psychosis, as well as for treatments that focus on social experiences and relationships.

## Supporting information

Heriot-Maitland et al. supplementary material Heriot-Maitland et al. supplementary material

## Supplementary material

The supplementary material for this article can be found at https://doi.org/10.1017/S0033291724001405.
