# Supplementary material for: Social influences on the relationship between dissociation and psychotic-like experiences
Source: Psychol Med. 2024 Jun 3;54(12):3285–93. doi: 10.1017/S0033291724001405 (PMC11496215; doi:10.1017/S0033291724001405)
Supplement: Heriot-Maitland et al. supplementary material [file S0033291724001405sup001.docx]

**Supplementary material**

**Appendix 1**

*Comparison of completers versus non-completers*

|  | **Completers**  (n=314) | | **Non-completers** (n=230) | | **Comparison**  **test** |
| --- | --- | --- | --- | --- | --- |
| **Gender**       Male       Female | 62  252 | (19.7%)  (80.3%) | 50  180 | (21.7%)  (78.3%) | *X*^2^(1, *N*=544) = .32,  *p*=.57 |
| **Age group**       Below 30       30 or over | 204  110 | (65.0%)  (35.0%) | 173  57 | (75.2%)  (24.8%) | *X*^2^(1, *N*=544) = 6.56,  ***p*=.01** |
| **Ethnicity**       White  Other | 253  61 | (80.6%)  (19.4%) | 159  71 | (69.1%)  (30.9%) | *X*^2^(5, *N*=544) = 9.46,  ***p*=.002** |
| **Language**       English       Other | 249  65 | (79.3%)  (20.7%) | 172  58 | (74.8%)  (25.2%) | *X*^2^(1, *N*=544) = 1.55,  *p*=.21 |
| **Education**       No degree       Degree or higher | 86  228 | (27.4%)  (72.6%) | 82  148 | (35.7%)  (64.3%) | *X*^2^(2, *N*=544) = 4.25,  ***p*=.04** |
| **MH services**       No       Yes | 180  134 | (57.3%)  (42.7%) | 150  80 | (65.2%)  (34.8%) | *X*^2^(1, *N*=544) = 3.47,  *p*=.06 |
| **DES**  *Mean (SD)* | 13.18 | (10.57) | 16.74 | (14.57) | *Z* = -2.38, ***p*<.05** |
| **TEQ**  *Mean (SD)* | 2.45 | (2.99) | 3.10 | (3.80) | *Z* = -1.07, *p*=.28 |
| **OAS**  *Mean (SD)* | 22.72 | (12.61) | 23.64 | (13.67) | *Z* = .873, *p*=.38 |
| **SSPS**  *Mean (SD)* | 37.75 | (8.78) | 37.87 | (8.97) | *Z* = -.56, *p*=.58 |

Significant results in **bold**
